# Short-Term Event Prediction in the Operating Room (STEP-OP) of Five-Minute Intraoperative Hypotension Using Hybrid Deep Learning: Retrospective Observational Study and Model Development

**DOI:** 10.2196/31311

**Published:** 2021-09-30

**Authors:** Sooho Choe, Eunjeong Park, Wooseok Shin, Bonah Koo, Dongjin Shin, Chulwoo Jung, Hyungchul Lee, Jeongmin Kim

**Affiliations:** 1 School of Medicine Yonsei University College of Medicine Seoul Republic of Korea; 2 Cerebro-Cardiovascular Research Institute Yonsei University College of Medicine Seoul Republic of Korea; 3 School of Industrial and Management Engineering Korea University Seoul Republic of Korea; 4 SK Inc C&C Seoul Republic of Korea; 5 School of Medicine Seoul National University College of Medicine Seoul Republic of Korea; 6 Department of Anesthesiology and Pain Medicine Seoul National University Seoul National University Hospital Seoul Republic of Korea; 7 Department of Anesthesiology and Pain Medicine Yonsei University College of Medicine Seoul Republic of Korea

**Keywords:** arterial pressure, artificial intelligence, biosignals, deep learning, hypotension, machine learning

## Abstract

**Background:**

Intraoperative hypotension has an adverse impact on postoperative outcomes. However, it is difficult to predict and treat intraoperative hypotension in advance according to individual clinical parameters.

**Objective:**

The aim of this study was to develop a prediction model to forecast 5-minute intraoperative hypotension based on the weighted average ensemble of individual neural networks, utilizing the biosignals recorded during noncardiac surgery.

**Methods:**

In this retrospective observational study, arterial waveforms were recorded during noncardiac operations performed between August 2016 and December 2019, at Seoul National University Hospital, Seoul, South Korea. We analyzed the arterial waveforms from the big data in the VitalDB repository of electronic health records. We defined 2s hypotension as the moving average of arterial pressure under 65 mmHg for 2 seconds, and intraoperative hypotensive events were defined when the 2s hypotension lasted for at least 60 seconds. We developed an artificial intelligence–enabled process, named short-term event prediction in the operating room (STEP-OP), for predicting short-term intraoperative hypotension.

**Results:**

The study was performed on 18,813 subjects undergoing noncardiac surgeries. Deep-learning algorithms (convolutional neural network [CNN] and recurrent neural network [RNN]) using raw waveforms as input showed greater area under the precision-recall curve (AUPRC) scores (0.698, 95% CI 0.690-0.705 and 0.706, 95% CI 0.698-0.715, respectively) than that of the logistic regression algorithm (0.673, 95% CI 0.665-0.682). STEP-OP performed better and had greater AUPRC values than those of the RNN and CNN algorithms (0.716, 95% CI 0.708-0.723).

**Conclusions:**

We developed STEP-OP as a weighted average of deep-learning models. STEP-OP predicts intraoperative hypotension more accurately than the CNN, RNN, and logistic regression models.

**Trial Registration:**

ClinicalTrials.gov NCT02914444; https://clinicaltrials.gov/ct2/show/NCT02914444.

## Introduction

Intraoperative hypotension due to low blood pressure during surgery may cause acute kidney injury, myocardial injury, and mortality [1,2]. Researchers have found evidence of a causal relationship between hypotension during surgery and organ dysfunction [3]. Therefore, reducing the frequency and duration of hypotension during surgery could prevent adverse postoperative outcomes. Intraoperative hypotension is defined as a mean arterial pressure <65 mmHg during surgery. Real-time prediction of hypotension may help anesthesiologists detect and intervene earlier during surgery, leading to a better prognosis. During surgery, the anesthesiologist interprets hemodynamic parameters, and immediately uses cardioactive drugs and fluid resuscitation to treat hypotension. However, it is difficult to predict the occurrence of hypotension through continuous intensive intraoperative monitoring.

Researchers have utilized various statistical methods, machine learning, and deep-learning techniques to predict hypotension [4-6]. In particular, the hypotension prediction index (HPI) is utilized as an on-the-shelf product based on high-fidelity arterial waveform data from the operating room (OR) [4]. The HPI uses the Flotrac algorithm to preprocess the arterial waveform and extract features for the logistic regression model.

Real-time automated data acquisition of multiple biosignals in the OR has facilitated the implementation of various deep-learning technologies to predict intraoperative events. For example, invasive arterial waveform-based convolutional neural network (CNN) has yielded remarkable results in intraoperative hypotension prediction [6] and stroke volume estimation [7]. Recurrent neural network (RNN) for time-series prediction has successfully predicted in-hospital cardiac arrest and respiratory failure [8,9] owing to the time-dependent nature of the biosignals [9,10].

A CNN consists of convolution layers and pooling layers; convolution layers filter input data to produce feature maps indicating the locations and strength of detected features in the input data, and pooling layers downsample the feature maps by summarizing the presence of features in patches of the feature map [11]. By contrast, RNNs are designed to process sequential inputs such as language or time-dependent signals. An RNN processes an input sequence one at a time, retaining information in a hidden state vector. Specifically, long short-term memory (LSTM) networks use special hidden units, which act as gated leaky neurons, thus remembering the input for a long time. LSTM networks are known to be more effective than conventional RNNs [11]. Both CNNs and RNNs can process signal data and are hence suitable for analyzing biosignals. A CNN focuses on specific patterns in the signal, whereas an RNN processes temporal information found in the sequences of the signals.

The logistic regression model has been outperformed by deep-learning models in terms of various medical applications, including in-hospital cardiac arrest prediction [9], aortic valve calcification prediction [12], and stroke prediction [13].

In this study, we propose the short-term event prediction in the operating room (STEP-OP) hypotension prediction system based on the weighted average ensemble of individual neural networks that utilizes biosignals recorded during noncardiac surgery. To this end, the arterial waveforms of 18,813 patients were selected, segmented, and labeled autonomously according to a criterion that enabled the construction and extension of deep-learning models with big data from real-time recording systems.

## Methods

### Overview

STEP-OP was developed to predict intraoperative hypotension 5 minutes before it occurs based on big data from the VitalDB [14] repository of electronic health records and intraoperative biosignals. The records were collected by VitalRecorder, a software for automatically recording time-synchronized physiological data, including arterial waveform and electrocardiogram data [15].

**Figure 1:**
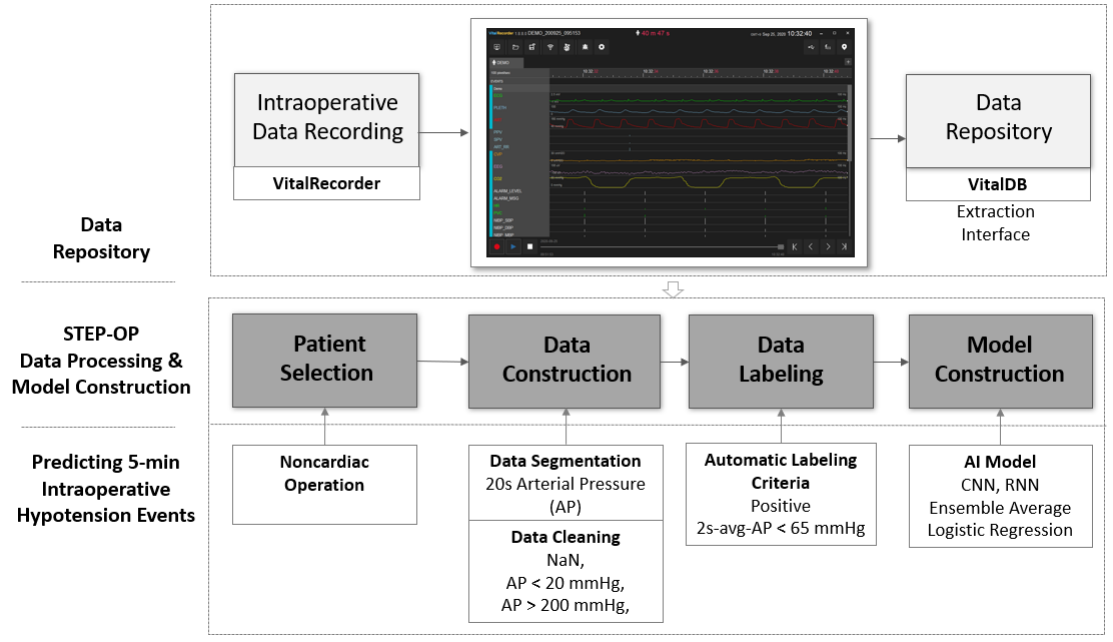
Process flow and criteria of short-term event prediction in the operating room (STEP-OP) for constructing the prediction model of intraoperative hypotension using VitalDB. CNN: convolutional neural network; RNN: recurrent neural network; NaN: missing values.

The process flow of STEP-OP consists of (i) patient selection, (ii) data construction with automatic segmentation of biosignals and data cleaning, (iii) automatic labeling, and (iv) construction of the prediction model (Figure 1). Details of each process are described below.

### Subject Selection

We selected all patients whose arterial waveforms were recorded during noncardiac operations performed between August 2016 and December 2019, at Seoul National University Hospital, Seoul, South Korea. A total of 21,321 patients were enrolled in this retrospective study for constructing the prediction model of intraoperative hypotension. The study was approved by the institutional review board of Seoul National University Hospital (H-2008-175-1152) and is registered at ClinicalTrials.gov (NCT02914444).

### Data Construction

The arterial waveforms were recorded at 100 or 500 Hz and were downsampled to 100 Hz. Each 60-second segment was observed paired with a 20-second segment that occurred 5 minutes previously.

To detect artifacts in the arterial waveforms, we excluded waveforms clearly beyond the physiological range according to the following criteria: (1) segments with missing values, (2) segments with blood pressure over 200 mmHg or under 20 mmHg, (3) segments with a difference between the maximum and minimum pressure value under 20 mmHg, and (4) segments with a difference between adjacent values over 30 mmHg (pressure gradient over 3000 mmHg/second). The 20- or 60-second segment of the arterial waveforms that met any of the criteria listed above was excluded from the dataset. No modifications were made to the extracted waveform segments.

**Figure 2:**
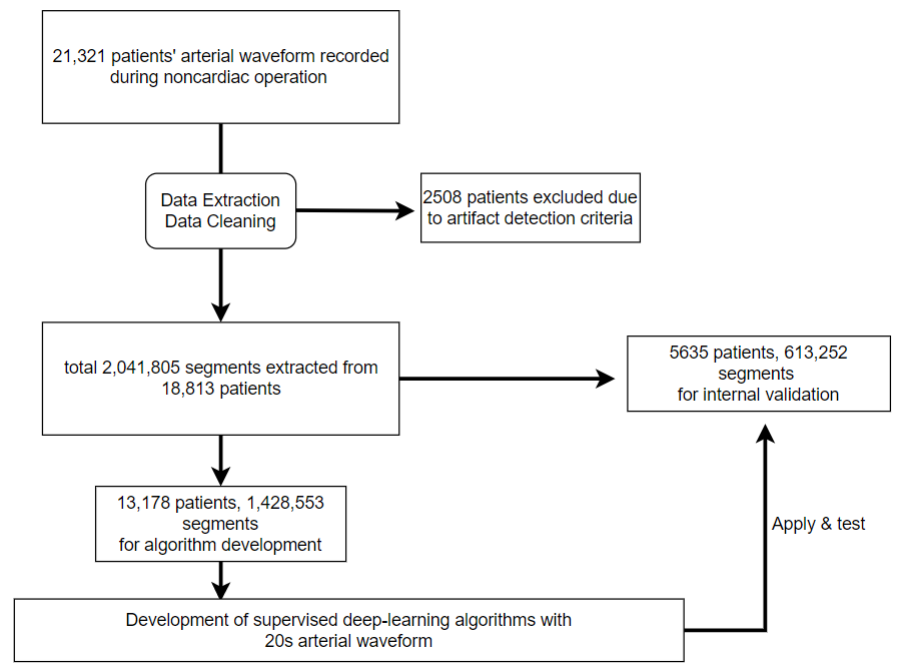
CONSORT diagram with flow of data construction.

Among the 21,321 patients, 2508 patients were excluded from the study after failing the data cleaning step according to the criteria. In total, the data segmentation process produced 2,041,805 segments from 18,813 patients. Patients were randomly split into 70/30 training and validation sets. Further, 1,428,553 segments from 13,178 patients’ data were used for algorithm development, and 613,252 segments from 5635 patients’ data were used for internal validation (Figure 2).

### Data Labeling

STEP-OP predicts hypotension 5 minutes before its onset based on 20-second arterial waveforms. First, we defined 2s hypotension as the moving average of arterial pressure under 65 mmHg for 2 seconds, and the intraoperative hypotensive event was defined as the case in which the 2s hypotension lasts for at least 60 seconds. Accordingly, 20-second segments of the arterial waveform 5 minutes before the event were selected and labeled “positive instances.” If the 2-second moving average was maintained over 75 mmHg for at least 60 seconds, it would be considered a nonhypotensive event. The 20-second segments 5 minutes before the onset were selected and labeled “negative.”

### Algorithm Development

**Figure 3:**
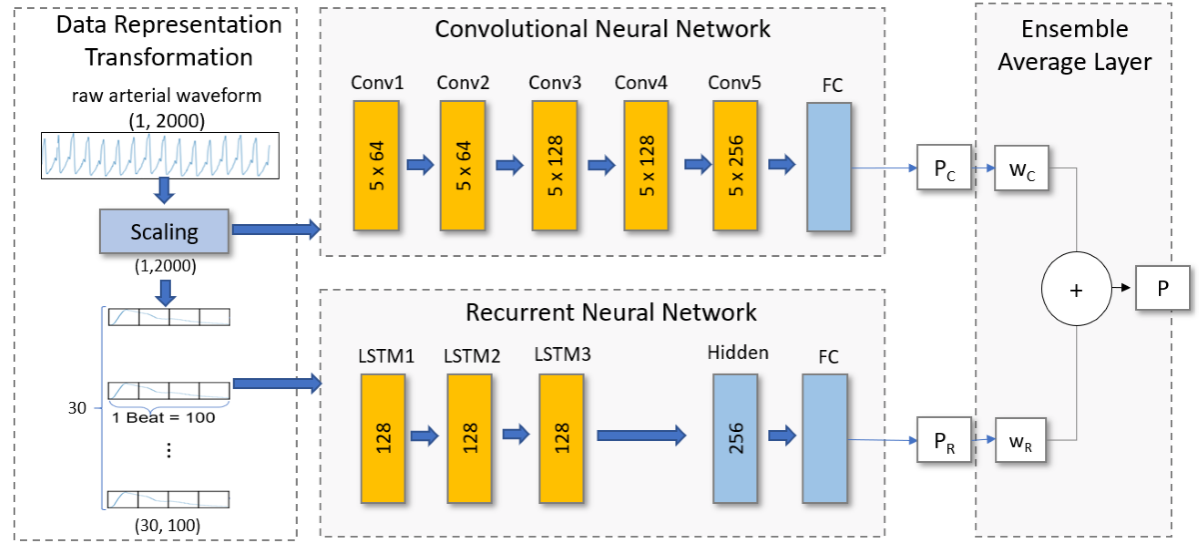
Short-term event prediction in the operating room (STEP-OP) model construction. K×F denotes the kernel size and number of filters. ReLU activation was used after each convolution layer, and the sigmoid was used for the final activation. Normalization, pooling, and dropout layers are omitted in the figure. LSTM: long short-term memory; FC: fully connected layer.

We developed an ensemble average model from two distinct deep-learning layers of a CNN and RNN. The combination of multiple neural networks can outperform individual networks while offering the advantage of generalization [16]. The combination of heterogeneous deep neural networks, especially CNNs and RNNs, has shown better performance in various applications [17,18]. Figure 3 depicts the process of STEP-OP model construction with data preprocessing and the ensemble average model of neural networks. The CNN is composed of 1D convolution, batch normalization, pooling, and fully connected layers for the input of scaled 20-second arterial pressure (array with a length of 2000). The RNN is composed of three stacked bidirectional LSTM networks for the input of 30×100 tensors derived from the scaled 20-second arterial waveform (array with a length of 2000). To preprocess the arterial waveform, the array was sliced into individual cardiac cycles using peak detection algorithms, and each cycle was interpolated to an array with a length of 100. Thus, each cardiac cycle represents a time step. If the 20-second segment had more or less than 30 time steps (cardiac cycles), it was pretruncated or prepadded with zero vectors, respectively (Figure 3).

The final stage of model construction computes the ensemble average prediction value, *P*(*α*)=*αP_RNN_*+(1–*α*)*P_CNN_,* where *α* is a weighting factor, and *P_RNN_* and *P_CNN_* are the output prediction values of the RNN and CNN, respectively. We derived *α* by evaluating the area under the precision-recall curve (AUPRC) of *P*(*α*) on 10% of the training data. Finally, the performance of the ensemble average was evaluated on the validation set using the *α* derived from 10% of the training data.

We used the Pytorch deep-learning framework [19], the AdamW optimizer with *β_1_*=0.9 and *β_2_*=0.99, and the binary cross entropy for the loss function with the learning rate set to 10^–4^. To prevent the model from being biased toward the majority class, losses with weights of 1 and 20 were used for the negative and positive data points, respectively. We chose the optimal hyperparameters by training deep-learning models on 90% of the training data and chose the models that performed the best on the remaining 10%.

We evaluated the performance of the proposed model by comparing it with the logistic regression model based on the feature set of 12 features from the 20-second arterial waveforms (Table 1).

**Table 1:** Features from the arterial waveform segments.

Feature symbol	Description
Mean_beat_length	Average time of cardiac cycle
MAP^a^	Average MAP of cardiac cycle
PP^b^_max	Maximum value among pulse pressure
PP_min	Minimum value among pulse pressure
PP_range	PP_max–PP_min
PP_avg	Average pulse pressure
PPV^c^	(PP_max–PP_min)×2.0/(PP_max+PP_min)
Systolic_time_avg	Average systolic time
Systolic_pressure_avg	Average systolic pressure
Systolic_pressure_range	Difference between maximum systolic pressure and minimum systolic pressure
Diastolic_pressure_avg	Average diastolic pressure
Beat_area_avg	Average of area under cardiac cycles

^a^MAP: mean arterial pressure.

^b^PP: pulse pressure.

^c^PPV: pulse pressure variation.

We performed robust scaling after extracting the features, as *x_i_*′=x*_i_*–Q*_i_*,_2_/*Q_i_*_,3_–*Q_i_*_,1_, where *x_i_*, *x_i_′*, and *Q_i,j_* denotes the value of the *i*th feature, the scaled value of the *i*th feature, and the *j*th quartile value of the *i*th feature, respectively.

The logistic regression model with five-fold cross-validation was implemented using scikit-learn [20].

The prediction models of an imbalanced dataset are evaluated in terms of the performance metrics AUPRC, area under the receiver operating characteristic curve, precision, and sensitivity (recall) since the negative data points significantly outnumbered positive data points [21,22]. Precision was evaluated at thresholds at which the sensitivity is 0.6, 0.7, and 0.8. For performance evaluation, we used the bootstrap method to estimate the 95% CI, resampling 50% of the dataset 1000 times randomly.

## Results

The proposed method helped us to select 18,813 patients for the study. The mean age of the group was 58.5 (SD 15.3) years. Approximately 49.3% of patients in the group were male. The training cohort (n=13,178) presented 1,373,378 negative segments and 55,175 positive segments (total 476,184 minutes). The validation cohort (n=5635) presented 587,413 negative segments and 25,839 positive segments (total 204,417 minutes). Table 2 compares the demographic characteristics between the training and validation cohorts.

Table 3 summarizes the statistical results of 12 features extracted from the arterial waveform segments.

**Table 2:** Study population characteristics.

Characteristic		Total	Training cohort	Validation cohort	*P* value
Number of patients		18,813	13,178	5635	N/A^a^
Age (years), mean (SD)		58.5 (15.3)	58.6 (15.2)	58.2 (15.4)	.13
Weight (kg), mean (SD)		63.4 (12.8)	63.5 (12.8)	63.4 (12.8)	.68
Height (cm), mean (SD)		162.2 (10.0)	162.2 (10.0)	162.4 (9.9)	.07
Male, n (%)		9270 (49.27)	6416 (48.69)	2854 (50.65)	.01
**ASA^b^ score, n (%)**					.47
	I	4352 (23.14)	3084 (23.40)	1268 (22.50)	
	II	11,428 (60.75)	7970 (60.48)	3458 (61.37)	
	III	2824 (15.01)	1980 (15.03)	844 (14.98)	
	IV	196 (1.04)	137 (1.04)	59 (1.05)	
	IV	13 (0.07)	7 (0.05)	6 (0.11)	

^a^N/A: not applicable.

^b^ASA: American Society of Anesthesiologists.

**Table 3:** Feature characteristics.

Feature symbol	Positive event, mean (SD)	Negative event, mean (SD)	*P* value
Mean_beat_length (s)	0.82 (0.19)	0.89 (0.17)	<.001
MAP^a^ (mmHg)	64 (12)	90 (12)	<.001
PP^b^_max (mmHg)	55 (16)	60 (15)	<.001
PP_min (mmHg)	45 (16)	50 (14)	<.001
PP_range (mmHg)	10.0 (10.7)	9.8 (9.8)	<.001
PP_avg (mmHg)	50 (15)	55 (14)	<.001
PPV^c^	0.23 (0.29)	0.20 (0.25)	<.001
Systolic_time_avg (s)	0.13 (0.04)	0.14 (0.04)	<.001
Systolic_pressure_avg (mmHg)	98 (19)	125 (18)	<.001
Systolic_pressure_range (mmHg)	10.7 (10.2)	10.4 (9.0)	<.001
Diastolic_pressure_avg (mmHg)	49 (10)	71 (11)	<.001
Beat_area_avg (mmHg×s)	53 (16)	79 (18)	<.001

^a^MAP: mean arterial pressure.

^b^PP: pulse pressure.

^c^PPV: pulse pressure variation.

**Figure 4:**
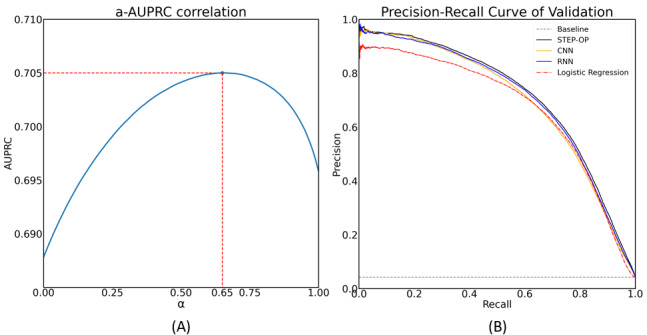
(A) Optimal weight value α on 10% of the training set. (B) Precision-recall curve of developed models. AUPRC: area under the precision-recall curve; CNN: convolutional neural network; RNN: recurrent neural network; STEP-OP: short-term event prediction in the operating room.

Figure 4 shows the configuration of the weight for the ensemble in STEP-OP and the corresponding performance of STEP-OP and other methods. Figure 4A shows the optimal weight value *α_max_*, which maximizes the AUPRC value. AUPRC reached its highest value when *α* was 0.65. Thus, we evaluated the ensemble average *P*_STEP-OP_=0.65×*P_RNN_*+0.35×*P_CNN_* on the validation set.

Figure 4B illustrates the performance of the prediction models with respect to the AUPRC. Deep-learning algorithms using raw waveform as input (CNN, RNN) achieved higher AUPRC scores than the logistic regression algorithm. STEP-OP obtained the best performance, with a higher AUPRC than either the CNN or RNN algorithm (see Table 4).

**Table 4:** Performance of each algorithm in the internal validation cohort.

Algorithm	AUPRC^a^ (95% CI)	AUROC^b^ (95% CI)	Sensitivity^c^ (95% CI)				Precision^c^ (95% CI)		
			0.6	0.7	0.8	0.6		0.7	0.8
STEP-OP^d^	0.716 (0.708-0.723)	0.961 (0.959-0.962)	0.600 (0.591-0.609)	0.700 (0.692-0.708)	0.800 (0.793-0.806)	0.742 (0.733-0.751)		0.647 (0.640-0.655)	0.502 (0.495-0.509)
Convolutional neural network	0.698 (0.690-0.705)	0.955 (0.953-0.957)	0.600 (0.591-0.608)	0.700 (0.692-0.708)	0.800 (0.793-0.806)	0.717 (0.709-0.726)		0.615 (0.606-0.622)	0.466 (0.459-0.472)
Recurrent neural network	0.706 (0.698-0.715)	0.958 (0.956-0.959)	0.600 (0.591-0.608)	0.700 (0.692-0.708)	0.800 (0.793-0.806)	0.738 (0.729-0.746)		0.639 (0.631-0.647)	0.488 (0.481-0.495)
Logistic regression	0.673 (0.665-0.682)	0.948 (0.946-0.950)	0.600 (0.592-0.609)	0.700 (0.691-0.708)	0.800 (0.793-0.807)	0.711 (0.703-0.720)		0.622 (0.614–0.630)	0.481 (0.474-0.487)

^a^AUPRC: area under the precision-recall curve.

^b^AUROC: area under the receiver operating characteristic curve.

^c^Sensitivity and precision values were evaluated at the thresholds for sensitivity of 0.6, 0.7, and 0.8.

^d^STEP-OP: short-term event prediction in the operating room.

**Figure 5:**
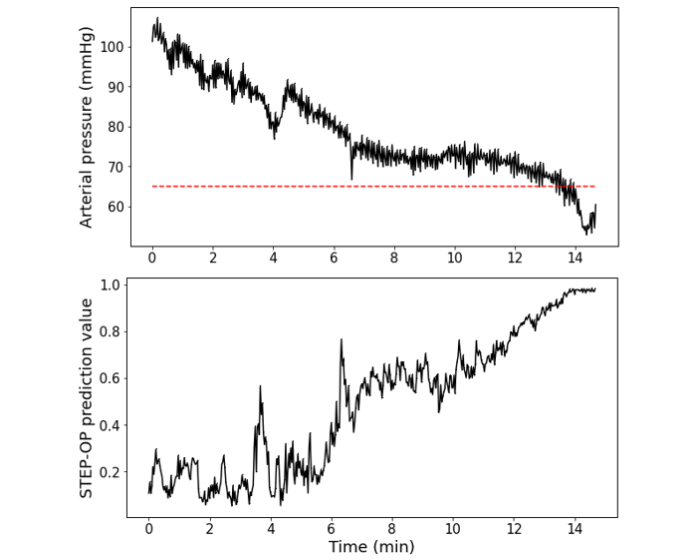
Example of a patient record depicting the arterial pressure and STEP-OP prediction values over time. Arterial pressure denotes the 2-second moving average of the arterial pressure. STEP-OP: short-term event prediction in the operating room.

Figure 5 illustrates the STEP-OP prediction values and arterial pressure of a validation cohort patient. Arterial pressure denotes the 2-second moving average of a waveform. After a gradual decrease, the patient’s arterial pressure stabilized around 75 mmHg. The pressure then plummeted abruptly, resulting in a hypotensive event at the 14th minute. The STEP-OP prediction scores were consistently above 0.6 from 7 minutes and started to increase from 9 minutes (ie, 5 minutes before the hypotensive event). Two minutes before the hypotensive event, STEP-OP prediction values peaked at over 0.8.

## Discussion

### Medical Artificial Intelligence Systems Utilizing Big Data

In this retrospective observational study, we developed deep-learning and machine-learning algorithms to predict an intraoperative hypotension event 5 minutes before its onset by leveraging a big data repository from an automatic recording system in the OR. Processing big data introduces several methodological challenges and opportunities in medical research [23]. We performed automatic data segmentation, cleaning, and labeling techniques on a large volume of biosignals according to the expert knowledge–based criteria of the target disease. By defining the hypotensive events and artifacts, we extracted several data points without manually labeling research data. This is essential for building artificial intelligence systems based on big medical data.

### Comparison With Previous Work

This study extends previous work on the HPI using an identical input of the high-fidelity 20-second arterial waveform. The HPI is the only algorithm currently used for predicting intraoperative hypotension [24]. It is based on a logistic regression and uses engineered features derived from the 20-second arterial waveform as input [4]. Other researchers have attempted to predict postinduction hypotension using either machine- or deep-learning technologies [5,25]. However, conventional machine-learning technologies require manually engineered features extracted from raw data because they lack the ability to process raw data [11]. Hence, the HPI requires separate preprocessing algorithms (eg, Flo-trac, CO-Trek) to calculate and process various features from the waveform [4]. In contrast, deep learning can automatically learn discriminative features from data [11]. The only preprocessing methods the CNN and RNN algorithms used in this study require are scaling and slicing.

Deep-learning algorithms may detect subtle changes in the arterial waveform, which predict sudden drops in the arterial pressure. These changes are likely to be masked or diminished when represented as features. As shown in Table 3, the CNN and RNN models using raw waveform data performed better than the logistic regression model using the calculated features.

Finally, the ensemble average of CNN and RNN predicted hypotension more accurately than each deep-learning model. In this study, the optimal weights for the ensemble of LSTM and CNN outputs were 0.65 and 0.35, respectively. This showcases the improved intraoperative hypotension prediction by the hybrid model STEP-OP over a single deep-learning model or logistic regression model.

### Limitations

This approach has a few limitations. First, we defined hypotension arbitrarily (2-second pressure moving average under 65 mmHg for hypotensive events, and 2-second moving average over 75 mmHg for nonhypotensive events). Prospective research must be performed to observe the effect of these criteria on the performance of the algorithms. Second, although a relatively large (N>10,000) cohort of patient data was used, it was retrieved from a single database. Future research will include external validations of different population distributions and settings. Finally, the threshold values and corresponding response of clinicians according to the STEP-OP prediction value must be determined for practical use in the OR. Prospective studies in actual clinical practice are needed to solve these limitations.

### Conclusion

We developed STEP-OP utilizing a big data repository and constructed a prediction model of short-term intraoperative hypotension. The weighted average of the deep-learning models performed the best in the prediction of hypotension. The proposed algorithms use only the 20-second arterial waveform without requiring separate feature computations. Consequently, they can be easily implemented in scenarios with the possibility of invasive blood pressure monitoring and can replace the HPI algorithm in those situations. The proposed solution can be extended and practically used for the real-time prediction of adverse events in the OR or intensive care units. This in turn is expected to improve clinical outcomes and reduce the burden of medical staff.

## Abbreviations

AUPRC: area under the precision-recall curve
CNN: convolutional neural network
HPI: hypotension prediction index
LSTM: long short-term memory network
OR: operating room
RNN: recurrent neural network
STEP-OP: short-term event prediction in the operating room
